# Burning Graphene Layer-by-Layer

**DOI:** 10.1038/srep11546

**Published:** 2015-06-23

**Authors:** Victor A. Ermakov, Andrei V. Alaferdov, Alfredo R. Vaz, Eric Perim, Pedro A. S. Autreto, Ricardo Paupitz, Douglas S. Galvao, Stanislav A. Moshkalev

**Affiliations:** 1 Center for Semiconductor Components, State University of Campinas, CP 6101, Campinas, SP, 13083-870, Brazil; 2 Instituto de Física “Gleb Wataghin”, Universidade Estadual de Campinas, 13083-970, Campinas, SP, Brazil; 3 Departamento de Física, IGCE, Universidade Estadual Paulista, UNESP, 13506-900, Rio Claro, SP, Brazil

## Abstract

Graphene, in single layer or multi-layer forms, holds great promise for future electronics and high-temperature applications. Resistance to oxidation, an important property for high-temperature applications, has not yet been extensively investigated. Controlled thinning of multi-layer graphene (MLG), e.g., by plasma or laser processing is another challenge, since the existing methods produce non-uniform thinning or introduce undesirable defects in the basal plane. We report here that heating to extremely high temperatures (exceeding 2000 K) and controllable layer-by-layer burning (thinning) can be achieved by low-power laser processing of suspended high-quality MLG in air in “cold-wall” reactor configuration. In contrast, localized laser heating of supported samples results in non-uniform graphene burning at much higher rates. Fully atomistic molecular dynamics simulations were also performed to reveal details of oxidation mechanisms leading to uniform layer-by-layer graphene gasification. The extraordinary resistance of MLG to oxidation paves the way to novel high-temperature applications as continuum light source or scaffolding material.

Graphene has been considered one of the most promising materials for generating novel technological applications in near future. This exciting material presents exceptional electrical^1^,^2^, optical^3^,^4^, mechanical^5^,^6^ and thermal^7^,^8^ properties. High thermal conductivity of graphene makes this material very attractive for heat management and dissipation in high-density devices, which is a serious issue in modern nanoelectronics.

Most of these studies have been so far focused on single layer graphene (SLG) structures. However, large-scale production of high quality SLG and its manipulation have remained technologically challenging^9^,^10^. On the other hand, few-layer and multi-layer graphene (FLG and MLG, respectively) have some interesting properties that distinguish them from the SLG form and can be exploited to create completely new applications^11^,^12^,^13^.

In particular, SLG is a flexible material that can be easily deformed when deposited on rough surfaces and it tends to fold up during processing in liquids, e.g., during transfer to other substrates^14^. This is one of the technological disadvantages of SLG that limits some of its applications. In contrast, with just a small number of layers, FLG becomes a much more rigid structure that maintains its flat shape and can be easily processed or transferred using liquid environments. Furthermore, FLG or MLG forms an atomically flat surface that can match perfectly to other flat surfaces, easily integrating with thermally grown silicon oxide, one of the basic materials in microelectronics. It is important to note that the quality of MLG flakes obtained by sonication from natural graphite can be quite high^15^, and various methods are currently under development that allow for gradual thinning of initially thicker samples. However, most of methods to produce thinner samples include processing by plasma or laser and heating in oxygen atmosphere that involve various processes of oxidation followed by gasification of graphitic layers^16^,^17^,^18^. Localized removal (patterning) of graphene layers is usually based on ablation by tightly focused high-power lasers^19^,^20^,^21^ or electron beam lithography patterning followed by etching with atomic oxygen^22^,^23^, with or without external heating. Oxidation under high temperature conditions was extensively studied for graphite^19^,^20^,^24^. It is generally believed that basal planes of graphite are nonreactive, with gasification mostly occurring on exposed graphitic edges, and oxidation within the basal planes occurring through defective sites where atomic oxygen adsorption is most likely. As a result, oxidative etching within the basal plane is much faster than that in the normal direction (axis c). Formation of defects within the basal plane and corresponding acceleration of the graphitic layers etching at temperatures exceeding 900 °C was observed^19^. However, most studies of graphite oxidation so far have been performed under conditions of “hot-wall” reactors when graphite samples and oxygen gas are in thermal equilibrium, in contrast to laser heating experiments where samples are heated locally, practically without heating of the environmental gas, so that the conditions of a “cold-wall” reactor are realized. Further, we will show that the differences in the processing conditions for these two cases have a dramatic effect on oxidation and gasification of graphitic layers.

Recently, we have observed^25^ that MLG structures (that can be also called thin graphite nanoplatelets for thickness exceeding 10 monolayers^15^) obtained by sonication of natural graphite demonstrate extremely high stability under heating by laser in air, with sample temperatures exceeding 1000 °C, i.e., under conditions when other graphitic materials including multi-walled carbon nanotubes (usually, more defective) are known to burn out very fast^26^,^27^.

Here, we further exploit these ideas comparing exposure of supported (deposited over solid substrates) and suspended MLG samples to localized laser heating in air. It is important that experiments were performed under cold-wall reactor conditions using a low-power (a few mW) laser focused at the sample, where only a small solid sample is heated by the laser, while the surrounding gas is maintained basically at room temperature. Low gas temperature implies extremely low dissociation degree^28^ and therefore low chemical reactivity of oxygen, as compared with conventional hot-wall reactors when both the solid support and surrounding gas are in thermal equilibrium. Under these conditions, as will be discussed below, MLG samples suspended over holey amorphous carbon grids can be heated by laser in air up to very high temperatures (T_MLG_ ~ 2000 K) without instantaneous burning out the samples. This strong heating is possible for suspended samples since the main heat losses in this case are due to heat conduction to air (<10^5^ W/m^2^ K)^25^, while losses due to heat conduction through the amorphous carbon material are estimated to be smaller than 10^−5^ W. Losses due to continuum radiation emission are much smaller, estimated to be ~10^−8^ W, so that T_MLG_ up to 2000 K or even higher can be achieved, for laser power of 1 mW and samples with surface area ~10 μm^2^.

Experimental configurations for laser heating of suspended and supported nanoplatelets are shown schematically in Fig. 1a,b, respectively. Suspended samples were obtained by deposition from MLG-containing solutions over commercial amorphous holey carbon grids used in transmission electron microscopy (TEM). The scanning electron microscope (SEM) images of samples before and after heating experiments are also shown. The results are remarkably different for suspended and supported samples. Suspended platelets (Fig. 1(a)) are etched mostly in-plane, resulting in a gradual and uniform thinning while the sample shape (lateral dimensions) is basically maintained. The laser heating experiments were performed using a confocal Raman spectrometer configuration, allowing for simultaneous monitoring of the characteristic Raman bands (e.g., G and D bands^15^). In particular, the frequency downshift of G band (alternatively, the ratio of anti-Stokes/Stokes components of the G band) is widely used for graphene temperature measurements, while the ratio of D/G bands intensities characterizes the material quality (i.e., presence of defects within the basal planes)^29^. When elevated laser power (>1 mW) was used, complete burning of the samples was observed to happen within a few seconds (usually, together with the supporting holey carbon grids), and we were able to observe the exact moment of the sample disappearance through the backscattered Raman signal. Gradual decrease of the Raman signal was observable for sample thicknesses smaller than 10 nm allowing avoiding the complete sample burning in order to obtain extremely thin (possibly, a few-layer) samples. In contrast, for samples supported over oxidized silicon substrates (Fig. 1(b)), there is a significant non-uniform cross-plane etching, eventually creating holes at the laser focus point with the diameter of near 0.5 μm. These holes can be etched through dozens of graphene layers (leaving just a few layers at the substrate, as the remaining graphene layers absorb a small fraction of the incident light and thus cannot be heated up to high temperatures^25^). Also, it was necessary to use higher laser powers (up to 10 mW) in order to etch supported samples.

The observed uniform and gradual thinning of suspended platelets under localized low-power laser heating is in striking contrast to most of reports published so far considering burning of graphitic materials exposed to air or oxygen under high temperatures, as well as to our experiments with supported samples. Extremely high thermal conductivity of high-quality graphitic materials (up to ~2000 W/m.K for MLG^8^) results in a fast heat redistribution over the graphitic surface. Furthermore, the absence of localized heat sinks for suspended nanoplatelets results in a uniform temperature distribution essential for uniform sample etching. Note that less uniform temperature distribution can be expected for samples supported over a solid silicon substrate where heat conductance to the substrate is mostly important. Finally, the high quality of samples (absence of point defects within the graphitic layers) is required for low cross-plane etching rate.

Note that for the hot-wall reactor configuration, high-energy oxygen atoms have been pointed out as responsible for formation of defects (vacancies) in graphitic sheets^30^,^31^,^32^, triggering the etching process. Nitrogen contribution to graphene etching is assumed to be much smaller, compared to those from oxygen^31^. In order to better understand the mechanisms of uniform thinning of suspended multi-layer graphene in our experiments, we have also performed extensive reactive molecular dynamics (MD) simulations of graphitic layers oxidation and gasification. To describe essential features of the experiments, we created a computational model in which a heated tri-layer graphene sample was exposed to an atmosphere composed of atomic oxygen. To mimic the cold-wall reactor experimental conditions, periodic boundary conditions were considered. In this way, the MD simulation results are contrasted below with the experimental ones. All molecular dynamics simulations were carried out using the ReaxFF force field what can effectively describe chemical reactions, in special combustion. This force field has been already applied with success for understanding of graphene oxidation^33^ process and materials in extreme environments^34^. Experimental data on etching rates within the basal plane (in-plane) and along the direction normal to the basal plane (cross-plane), measured in the present study and in other works^19^,^35^,^36^,^37^,^38^,^39^,^40^, are presented in Fig. 2. Data from literature are related to oxidation of graphitic samples (graphene or graphite) in furnaces (i.e., under conditions of hot-wall reactors), mostly for in-plane etch rate, whereas the cross-plane etch rate is expected to be much smaller. Thicknesses of suspended samples before and after laser irradiation were estimated here using tilted SEM images, and the temperatures of samples were estimated in-situ from the Raman spectra (see Methods for details).

The temperatures estimated from the Raman spectra are surprisingly high, up to 2000 K or even more. On the other hand, we also performed radiation spectra measurements in visible and near-infrared spectral region that confirmed strong increase of the radiation background corresponding to very high MLG surface temperatures (see Fig. 1S, Supplementary Materials), and the increase of the background intensity with increasing laser power was found to be consistent with the sample temperatures as estimated here from the Raman spectra.

As can be seen from Fig. 2(I), for suspended samples the values of both in-plane and cross-plane etch rates comparable with those reported in other works are achieved here at much higher temperatures (the temperature difference is ~500–750 K). In other words, suspended MLG samples appear much more stable against burning in air under high-temperature heating by laser. For suspended MLG flakes the cross-plane etching rate is at least one order of magnitude smaller than the in-plane one for temperature near 1250 K. For higher temperatures, the difference between the in-plane and cross-plane rates is gradually reduced. For supported samples, our results are consistent with some data reported in literature^19^,^40^ while in other cases^35^ the existing difference can be attributed to higher quality of our samples. Another important point is the absence of the D peak in Raman spectra for suspended samples (Fig. 2(II)). Since the D peak is associated with the level of disorder^29^, this means a very low defect density in the adjacent graphene layers during the Raman spectra acquisition time (usually, 1 sec). Since the in-plane etching rates are relatively high, this suggests that each layer, once damaged, is rapidly burned out, otherwise the D peak would appear in the spectra. In contrast, for supported samples where holes are etched in MLG during the irradiation, the D/G ratio is increasing steadily during the process (Fig. 2(II)).

In Fig. 3 we present the number of carbon-carbon bonds for each layer obtained from MD simulations. It is important to note that even though the conditions of simulations (see Methods for details) are significantly different from those for the real experiment (basically, oxygen atoms as oxidation agents instead of mostly molecular oxygen, respectively), the results basically confirm main tendencies observed in the experiment. As can be seen, when the first layer counting significantly drops (indicating the beginning of its effective etching), the count for the second one remains unchanged, meaning that the first layer is being etched while the second layer is still intact (see also Fig. 3). The count for the second layer starts to drop only when the count for the first layer almost reaches zero. The detailed process of graphene etching can be seen in Fig. 4, where we show representative snapshots from MD simulations. A video showing the whole simulation is included as part of Supplementary information.

MD simulations show that the etching process starts when atomic oxygen binds to a graphene layer forming epoxy structures. The presence of the epoxy groups allows carbon atoms to be extracted when more atomic oxygen reacts with the group, forming vacancies. These observations are in excellent agreement with the ones reported by Duin and co-workers^33^, which show that graphene breakup occurs in two stages: epoxide formation followed by the creation and growth of defects. The presence of defects in the layer surface greatly increases its reactivity causing fast in-plane etching. Higher reactivity of defective regions is a key ingredient to understand why the top layer is almost completely etched before the next, non-defective one, starts being burned. These combined (SEM, Raman and MD simulations) results strongly support our hypothesis that once one layer starts getting etched it is completely burned out before the next one starts.

It is important to stress that different gasification regimes are observed at different temperature ranges. Carbon dioxide formation is favored at low temperatures, characterizing a complete burning process. As the temperature reaches higher values, carbon monoxide formation becomes favored and carbon dioxide levels decrease. At this point, the formation of carbon trioxide can also be observed, but CO_3_ levels are kept at very small values and tend to zero as temperature reaches even higher values. For the highest temperature values analyzed, CO formation is once again favored causing a drop on the CO_2_ levels, as the burning process becomes considerably faster as seen in Fig. 3. Typical snapshots from MD simulations showing these different structures are presented in Fig. 4.

The reasons for the extraordinary oxidation resistance observed here in the suspended multi-layer graphene and burning under high-temperature laser heating in air are due to differences between our set-up and the ones usually employed in hot-wall reactor experiments^16^. First of all, extremely high quality graphene platelets are needed, as the presence of defects significantly lowers the reaction barriers, meaning the MLG would burn at much lower temperatures, as normally observed. Further, under the cold-wall reactor conditions, the oxygen gas in maintained basically at a room temperature so that the configuration “hot surface/cold gas” is realized. This makes huge difference with the case of a hot-wall reactor (“hot surface/hot gas”) where the edges of graphitic layers exposed to the high temperature gas can be etched simultaneously and very fast (this was confirmed also in our simulations). The last, and probably the most important point is to have MLG samples suspended thus providing practical absence of physical (thermal and electrical) contacts with supporting bulk solid materials. Note that the present set-up also eliminates possible supply and effects of impurities that are always present on supporting materials.

In summary, we presented a novel approach for processing of suspended high quality multi-layer graphene platelets by irradiation using focused low-power laser in air. For comparison, processing of supported MLG was also studied. The gas was kept at room temperature due to the “cold-wall” reactor set-up. Irradiation by laser was performed using a confocal Raman spectroscopy configuration allowing for simultaneous monitoring of the temperature and quality of samples. We have shown that laser heating results in very high temperatures (up to 2000 K or even more) and the burning process happens in completely different ways for suspended and supported samples. When MLG flakes were supported on a solid (oxidized silicon) substrate, holes through many layers are formed in the heated region. However, for suspended graphene samples, a uniform thinning through selective layer-by-layer etching has been achieved, without significant changes in lateral dimensions of the samples. The analysis of data obtained using Raman spectroscopy during processing of suspended samples indicates also that formation of initial defects within the basal planes exposed to gas is followed immediately by their complete removal through fast in-plane etching. In other words, under the present hot-sample/cold-gas conditions the time required for the formation of initial defects in a basal plane is much larger than that required for burning out of a damaged graphitic layer. Reactive molecular dynamics simulations were used to gain further insights on these processes and are fully consistent with these interpretations.

We have shown that with using a simple technique it is possible to controllably burn graphene layer by layer, thus allowing to obtain few layers or even single layer graphene. We would like to remark that using suspended multi-layer graphene samples (no thermal or electrical contacts with a solid substrate needed) as proposed here, two challenging graphene problems can be solved: high quality controlled thinning of multi-layer graphene and achieving high graphene temperatures, even when exposing samples to atmospheric air. The results can provide new ways to achieve even stronger heating of multi-layer graphene in vacuum or in different controlled gas environments. The new methodology presented here can be adapted to the fabrication of few and multi-layer graphene of controlled thickness with remarkable low defect density. At the same time it paves the way for a new series of graphene applications, such as continuous radiation source and/or as scaffolding material stable at extremely high temperatures. We hope the present work will stimulate further investigations along these lines.

## Methods

### Experiment

MLG flakes were produced from high quality natural graphite (Nacional de Grafite Ltda, Brazil) using mild ultrasound processing (sonication) in N,N-dimethylformamide (DMF) solutions^41^. Statistical study carried out by optical and SEM showed that most platelets have lateral dimensions in the range from 1 to 10 μm, and thickness from 10 to 100 nm, with the aspect ratio (lateral size/thickness) ranging from 50 to 300. Flakes were deposited over both standard amorphous holey carbon grids and Si substrates by drop casting. The quality of graphene was characterized via the confocal micro-Raman technique (NT-MDT) using 473 nm wavelength laser with power up to 10 mW and laser spot of 500 nm.

The gradual and uniform thinning of samples was confirmed in a large number (>30) of experiments with different sizes of samples (typically, 2–5 μm wide and from 10 to 100 nm thick), applied laser power (from 0.1 to 1 mW) and exposure time (from a few seconds to 6 hours, depending on the laser power).

**Figure 1:**
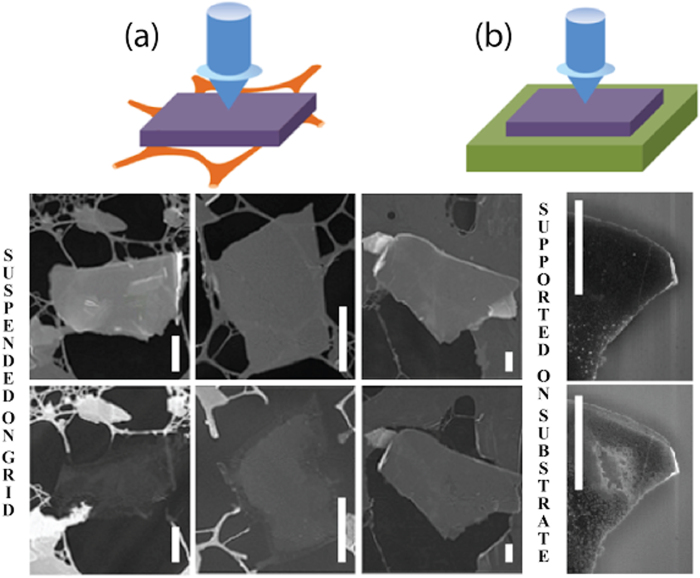
Experimental setup and SEM images for: suspended (**a**) and supported (**b**) multi-layer graphene (MLG) samples heated up by a focused laser beam. The corresponding SEM images correspond to samples before (top) and after (bottom) laser exposures, respectively. Suspended MLG samples are uniformly thinned, while supported MLG samples are etched non-uniformly, forming holes down to the substrate at the laser spot (0.5 μm in diam.). Scale bars –1 μm.

**Figure 2:**
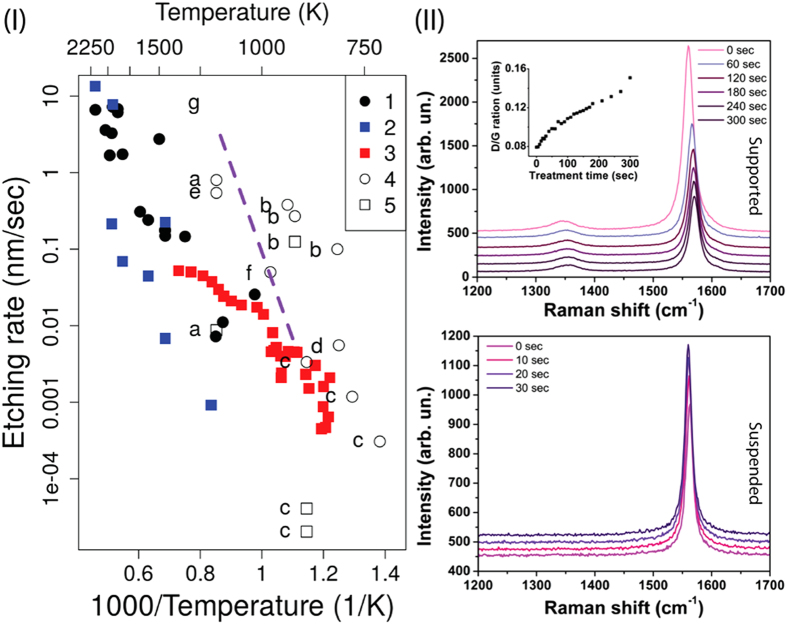
(**I**) Arrhenius plot for the temperature dependences of etching rates (1–3 – present study, 4,5 – other works): 1 – in-plane (suspended platelets), 2 – cross-plane (suspended platelets), 3 – cross-plane (supported platelets), 4 – in-plane (supported), 5 – cross-plane (supported); experimental data from other works: a^19^, b^35^, c^36^, d^37^, e^38^, f^39^, g^40^. In-plane etching represents the MLG flake etching along the basal plane (lateral size reduction), while cross-plane etching represents the etching in direction normal to the basal plane (thinning). (**II**) Raman intensities for G and D bands for suspended (bottom) and supported (top) graphene flakes during the laser exposure. The absence of the D peak in the suspended flakes spectra throughout the whole etching process demonstrates the high quality of the samples. In the case of supported flake the permanently increasing intensity of the D peak is a consequence of strong cross-plane etching and formation of hole.

From Fig. 2(I), it can be seen that temperatures as high as 2000 K were achieved in our laser heating experiments. The local sample temperatures were estimated using two methods: (i) from the Raman G band downshift^42^ and (ii) from the ratio of Stokes and anti-Stokes components of G band^43^. Note that for the G band frequency downshift method, the conversion coefficients were calibrated experimentally for relatively low temperatures (100–450 K)^42^. For multi-layer graphene, the conversion coefficient of −0.011 cm^−1^/K was measured, while for single layer and bi-layer graphene higher values were obtained (−0.016 cm^−1^/K and −0.015 cm^−1^/K). No data are available in literature for conversion coefficients at higher temperatures. Maximum G band downshift values observed here were as high as 30 cm^−1^, corresponding to near 3000 K or 2000 K maximum sample temperatures if −0.011 cm^−1^/K or −0.016 cm^−1^/K conversion coefficients (as for MLG or SLG, respectively) are used. Alternative method of graphene temperature determination is based on comparison of intensities for Stokes and anti-Stokes components of the G band^43^. Estimates using the last method gave maximum temperature values closer to 2000 K, as plotted in Fig. 2. Note that we also performed radiation spectra measurements in visible and near-infrared spectral region that confirmed strong increase of the radiation background corresponding to very high MLG surface temperatures (Fig. 1S, Supplementary information), and the increase of the background intensity with increasing laser power was found to be consistent with the sample temperatures as estimated here from the Raman spectra.

In heating experiments, we usually kept the laser power below 1 mW. When elevated laser power (>1 mW) was used, samples were observed completely burned out in a few seconds (usually, together with the supporting holey carbon grids), and we were able to observe the exact moment of the sample disappearance through the backscattered Raman signal. Gradual decrease of the Raman bands was evident for thickness of samples smaller than 10 nm allowing avoiding the complete sample burning.

To characterize changes in the shape and thickness of MLG platelets we used high resolution SEM images (200 Nanolab, FEI Co.) taken before and after heating procedure. For MLG flakes deposited onto Si-substrate we also made AFM (atomic force microscopy) measurements (NT MDT) to see profile change at the place where laser beam was focused.

We processed a large number of suspended samples, and measurable changes in the thickness and/or lateral size were detected for 24 of them (some of flakes were burnt out completely or fell down as a result of processing). Significant decreases of the surface area and of the thickness during the heating procedure were observed and measured for 18 and 8 flakes, respectively.

For the supported flakes, the in-plane etching rates were calculated by estimating the change on the area before and after laser heating from SEM images, normalizing it by the average perimeter of the flake and then dividing this value by the time of laser heating. The cross-plane etching rate was calculated by dividing the thickness change by treatment time. For the supported flakes the cross-plane etching rate was calculated following the approach proposed by Pop *et al.*^44^.

### Fully atomistic reactive molecular dynamics simulations

Our simulations were carried out considering trilayer graphene sheets (around 2500 A^2^) exposed to an atomic oxygen atmosphere (see Fig. 2S-10S, Supplementary information, for details). In order to mimic the few layers graphene structures, we kept fixed the first sheet. “Cold-wall” reactor conditions were simulated considering periodic boundary conditions, which protected borders from high oxidation rates. A temperature range (800 K–3000 K) was implemented in our simulations and controlled via a Nose-Hoover thermostat.

All molecular dynamics simulations were carried out using the ReaxFF^45^ force field as implemented on LAMMPS (large scale atomic molecular massive parallel simulator)^46^. The specific ReaxFF parametrization used during this work have been specifically tailored to describe combustion phenomena thus it is perfectly suited for the study of this problem^45^,^46^,^47^,^48^. Typical molecular dynamics considered a timestep of 0.1 fs and a total simulation time of ~150 ps.

## Additional Information

**How to cite this article**: Ermakov, V. A. *et al.* Burning Graphene Layer-by-Layer. *Sci. Rep.* **5**, 11546; doi: 10.1038/srep11546 (2015).

## Supplementary Material

Supplementary Information

Supplementary Movie S1

## Figures and Tables

**Figure 3:**
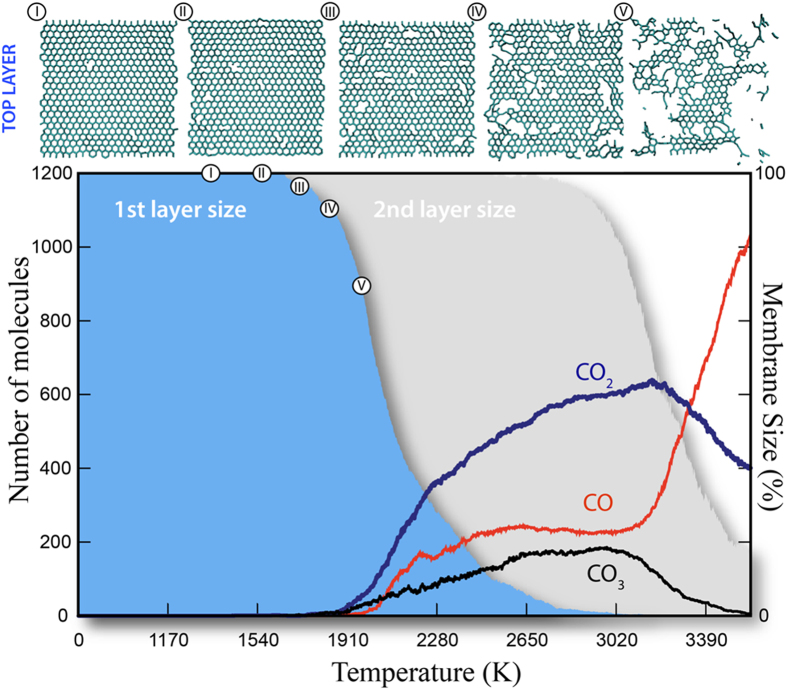
Bottom: Generated gas species during tri- layers graphene burning. First layer has been burned mainly via a complete burning process (CO_2_ formation). Second layer, under higher temperature, burned via an incomplete burning process (increasing contribution of CO and CO_3_ formation). Top: Snapshots showing evolution of the top graphitic layer during its burning.

**Figure 4:**
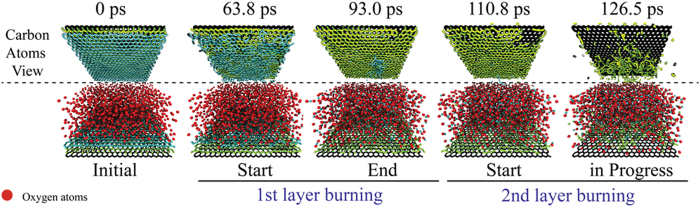
(**a**) Top and (**b**) side view of representative snapshots of the atomistic simulations. In (**a**) the oxygen (red) atoms are omitted for clarity. From these snapshots one can see that oxygen starts burning the second layer only after the first one is almost completely consumed.
