# Supplementary material for: Burning Graphene Layer-by-Layer
Source: Sci Rep. 2015 Jun 23;5:11546. doi: 10.1038/srep11546 (PMC4477407; doi:10.1038/srep11546)
Supplement: Supplementary Information [file srep11546-s1.pdf]

Supplementary information
Burning Graphene Layer-by-Layer

Victor A. Ermakov[1], Andrei V. Alaferdov[1], Alfredo R. Vaz[1], Eric Perim[2], Pedro A. S. Autreto[2], Ricardo Paupitz[3], Douglas S. Galvao[2] and Stanislav A. Moshkalev*[1]

[1] Center for Semiconductor Components, State University of Campinas, CP 6101, Campinas, SP, 13083-870, Brazil.
[2] Instituto de Física “Gleb Wataghin”, Universidade Estadual de Campinas, 13083-970, Campinas, SP, Brazil.
[3]Departamento de Física, IGCE, Universidade Estadual Paulista, UNESP, 13506-900, Rio Claro, SP, Brazil.

Experimental

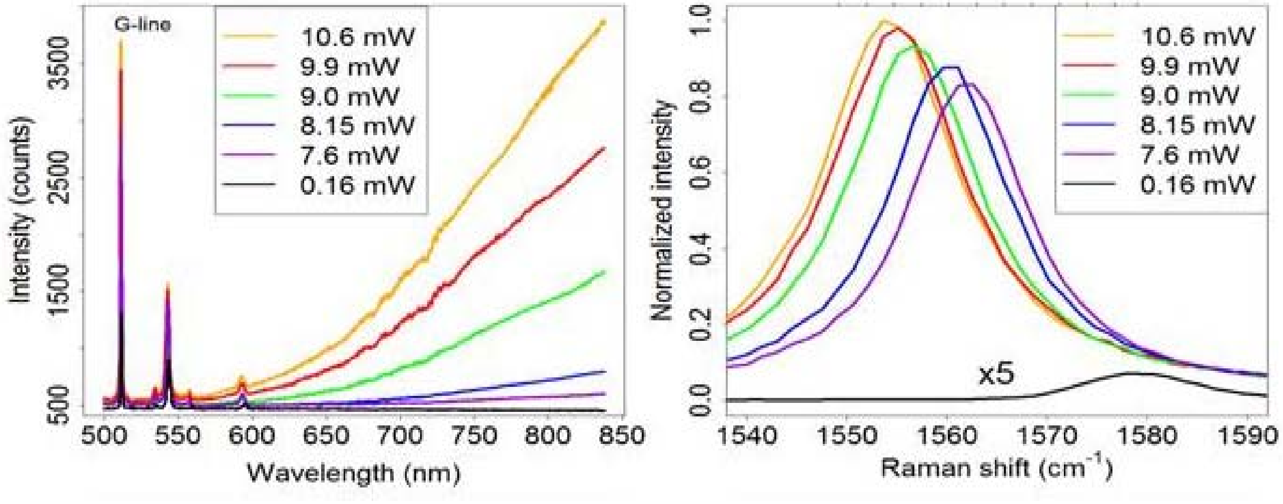

Fig. 1S. Left: Spectra of continuum emission from heated MLG platelets together with Raman peaks, for increasing laser power . Right: Downshift of Raman G peaks with increasing laser power. Note that the rise of Raman peak intensity (right) is proportional to the laser power, while the continuum intensity (left) grows much faster.

MODELING

SYSTEM

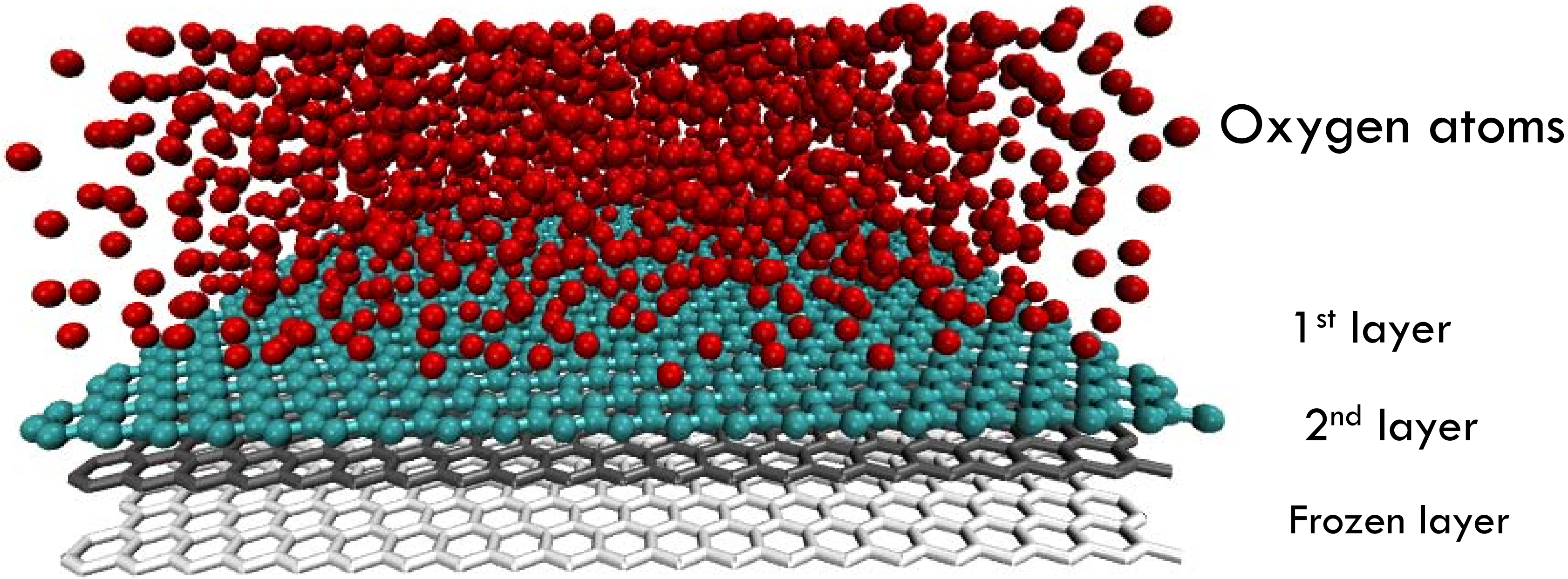

Fig. 2S.

MODELING

METHODOLOGY

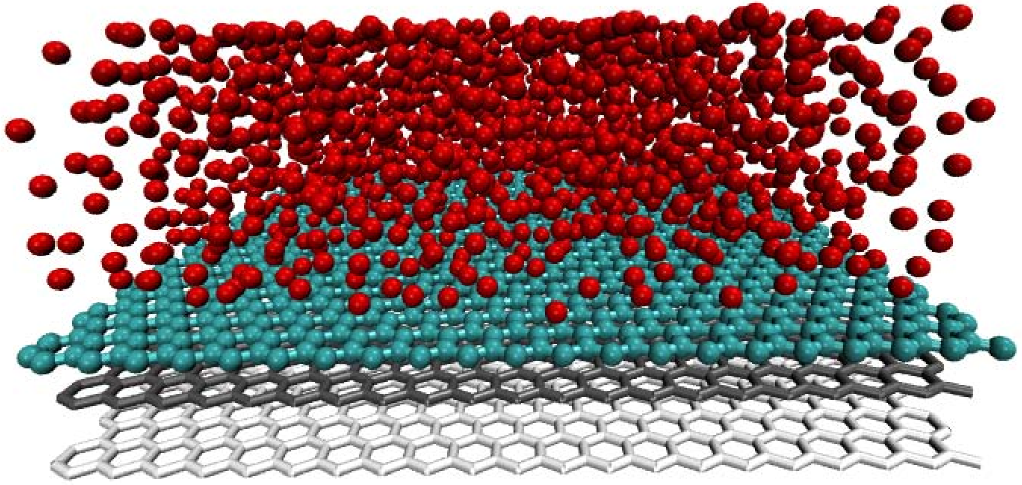

Periodic boundary conditions (PBC)

Fig. 3S

Settings

Method: Reactive Molecular Dynamics
Force field: ReaxFF
Thermostat: NVT
Temperature range: 800K-3500K
Timestep: 0.01 fs
Time: 150 ps

PROTECTING EDGES

METHOD (BEBO^2)

Implemented on	LAMMPS – Large-scale Atomic/Molecular Massively Parallel Simulator
Force Field	REAXFF[1,2]
Description	REAXFF is a BEBO^2 method. This empirical method allows the simulation of many types of chemical reactions, including bond dissociation

Fig. 4S

@2013 UNICAMP - ORGANIC SOLIDS AND NEW MATERIALS GROUP - AUTRETO

MOLECULAR DYNAMICS (MD)

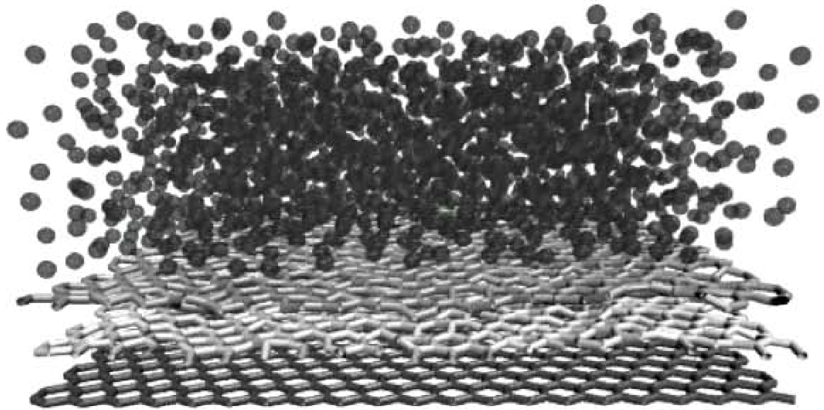

Fig. 5S.

MD – SNAPSHOTS

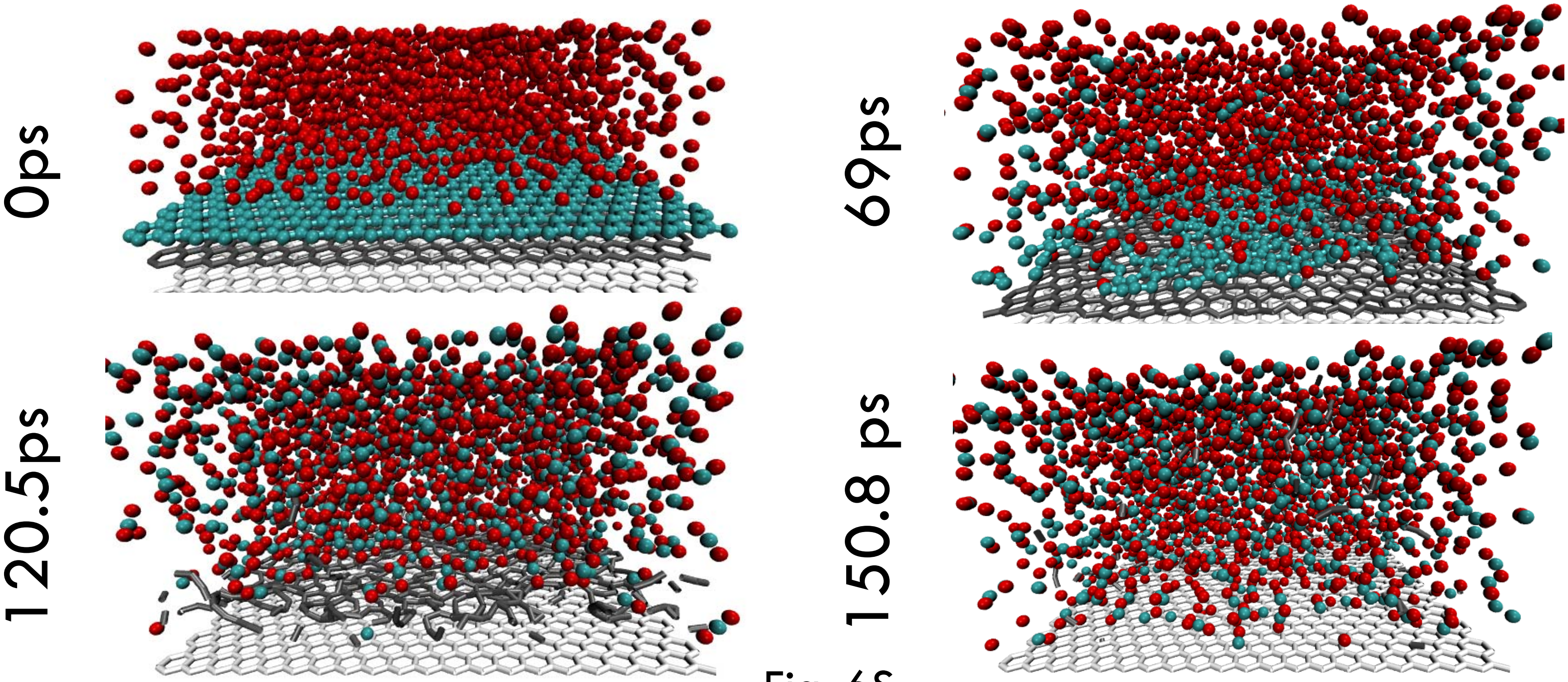

Fig. 6S

RESULTS

MD – TOP VIEW

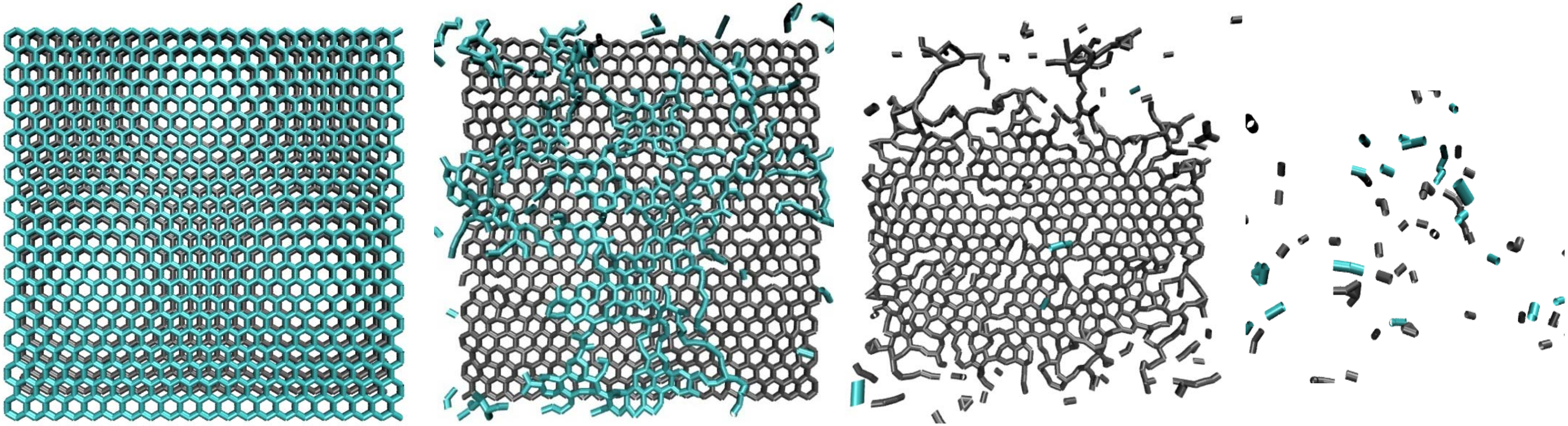

0ps 69ps 120.5ps 150.8 ps

Fig. 7S

MD – BURNING STEPS

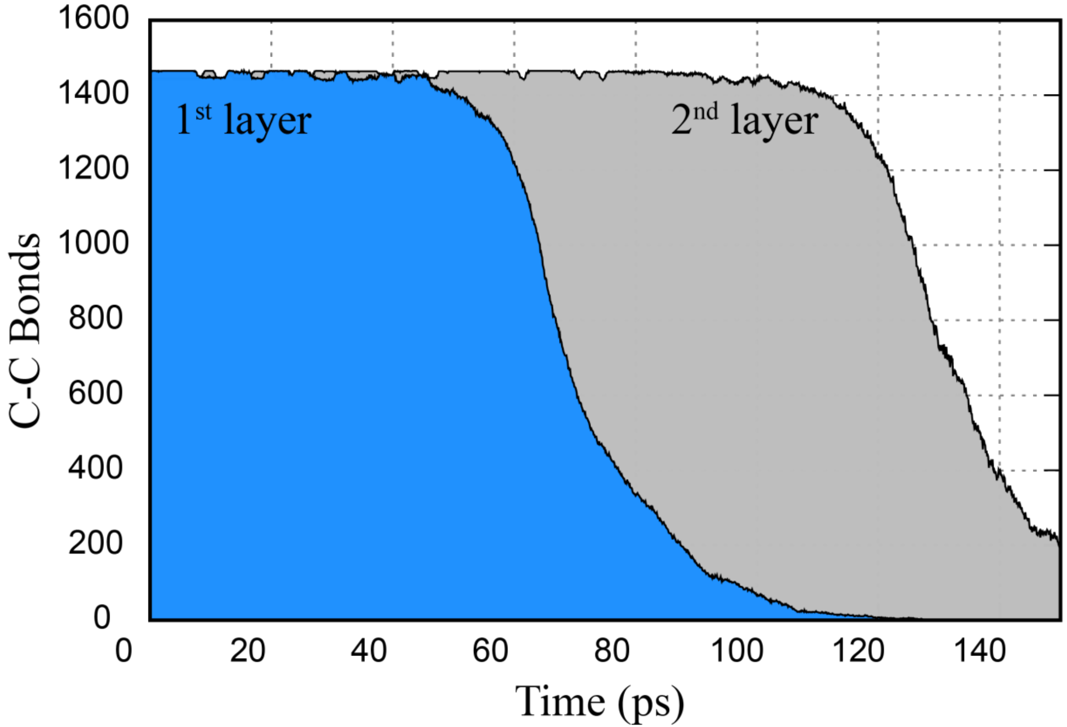

Fig. 8S

BURNING OCCURS "PER LAYERS".

See also the Supplementary video that presents the results of MD simulations of tri-layer graphene burning by oxygen atoms demonstrating clearly the sequential removal of layers.

MD – BURNING STEPS

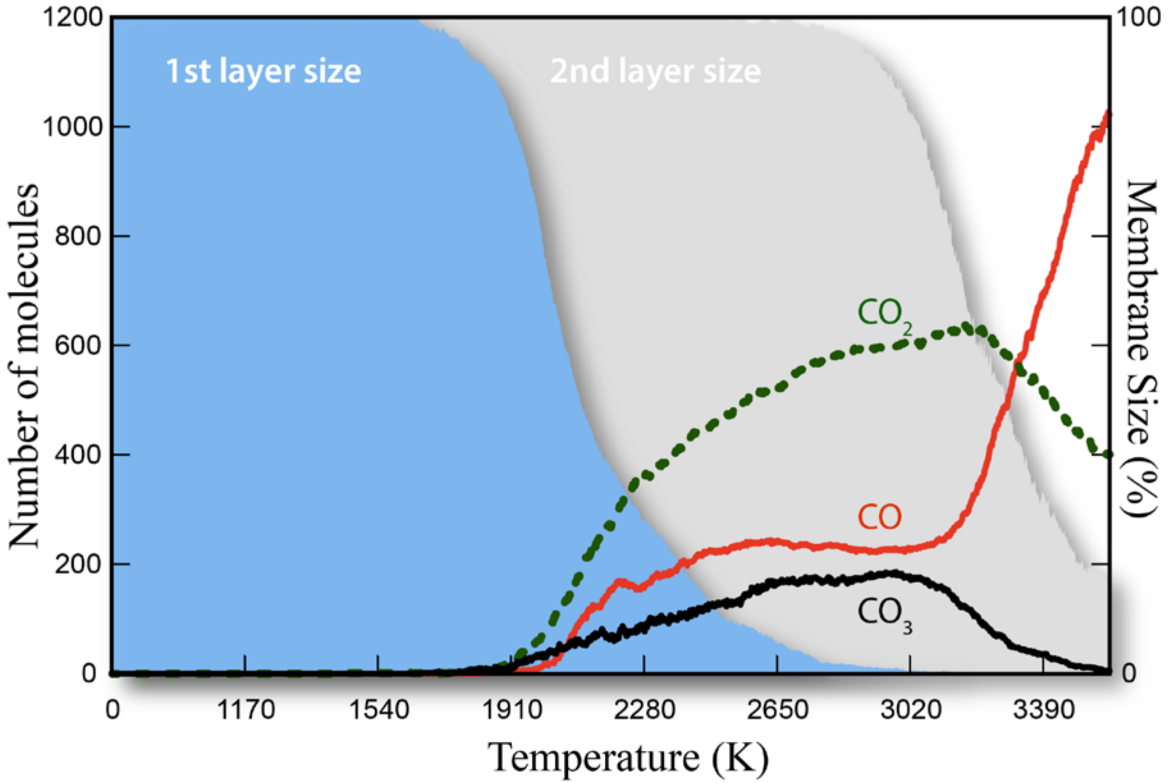

- <2500K: 1st layer burning - complete combustion (CO_2 formation)
- >3000K - 2nd layer burning – incomplete combustion (CO formation)

Fig. 9S

CONCLUSIONS

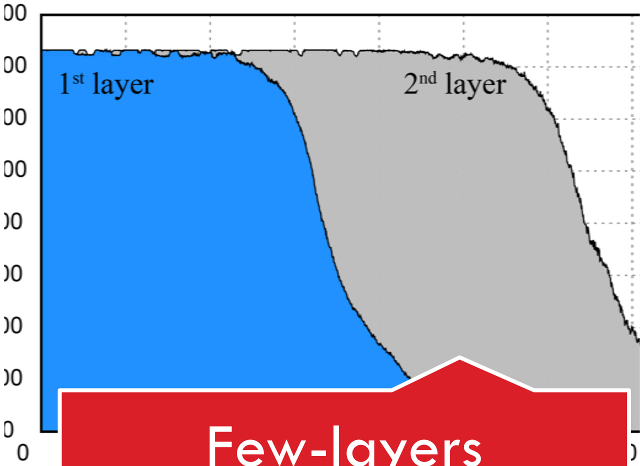

Few-layers graphene burns up by layers by formation of CO_2

Fig. 10S
